# Functional Mechanisms of Treg in the Context of HIV Infection and the Janus Face of Immune Suppression

**DOI:** 10.3389/fimmu.2016.00192

**Published:** 2016-05-19

**Authors:** Jacobo López-Abente, Rafael Correa-Rocha, Marjorie Pion

**Affiliations:** ^1^Laboratory of Immunoregulation, “Gregorio Marañón” Health Research Institute (IISGM), Madrid, Spain

**Keywords:** Treg cells, HIV Infections, regulatory mechanisms, IL-2, immune hyperactivation

## Abstract

Regulatory T cells (Tregs) play an important role in infections, by modulating host immune responses and avoiding the overreactive immunity that in the case of human immunodeficiency virus (HIV) infection leads to a marked erosion and deregulation of the entire immune system. Therefore, the suppressive function of Treg in HIV-infected patients is critical because of their implication on preventing the immune hyperactivation, even though it could also have a detrimental effect by suppressing HIV-specific immune responses. In recent years, several studies have shown that HIV-1 can directly infect Treg, disturbing their phenotype and suppressive capacity via different mechanisms. These effects include Foxp3 and CD25 downregulation, and the impairment of suppressive capacity. This review describes the functional mechanisms of Treg to modulate immune activation during HIV infection, and how such control is no longer fine-tune orchestrated once Treg itself get infected. We will review the current knowledge about the HIV effects on the Treg cytokine expression, on pathways implying the participation of different ectoenzymes (i.e., CD39/CD73 axis), transcription factors (ICER), and lastly on cyclic adenosine monophosphate (cAMP), one of the keystones in Treg-suppressive function. To define which are the HIV effects upon these regulatory mechanisms is crucial not only for the comprehension of immune deregulation in HIV-infected patients but also for the correct understanding of the role of Tregs in HIV infection.

## Introduction

Human immunodeficiency virus (HIV) infection is hallmarked by a depletion of CD4+ T-cell pool and a persistent immune activation that may lead to a progressive degradation and senescence of the immune system (1, 2). Chronic immune activation is considered at present as a better predictor of HIV-1 disease progression than the extent of viral replication (2, 3). Furthermore, activated CD4+ T cells constitute the main target of HIV infection and its subsequent active replication (4); therefore, the onset of systemic immune activation will be favorable for the HIV spreading and viral persistence. One of the keystones responsible for preventing such immune hyperactivation and controlling immune cell proliferation are regulatory T cells (Tregs). Tregs are a subset of CD4+ T cells characterized by the high surface expression of the high-affinity interleukin 2 receptor alpha (CD25 or IL-2-Rα) and the nuclear transcription factor forkhead box P3 (Foxp3) (5–7). In the majority of infectious episodes, Tregs get activated and, thus, induced to proliferate in order to control overreactive immunity thanks to their suppressive capacity (8). Their suppressive function is achieved by several suppressor mechanisms, including disruption of metabolic pathways (CD39/CD73 axis, cyclic adenosine monophosphate (cAMP), and ICER pathways) (9–13), modulation of APC maturation and function (CTLA-4 and PD1/PD-L1-dependent tolerogenic APC induction) (14–19), production of anti-inflammatory cytokines (IL-10, TGF-β, and IL-35) (20–22), and induction of apoptosis (via Fas/Fas-ligand pathway, granzyme A/B and perforin, TRAIL, the galectin-9/TIM-3 pathway, or galectin-1 production) (23–27). Tregs can suppress immune responses and, therefore, limit collateral tissue injury; however, their function could be also favorable for pathogen persistence by suppressing antigen-specific T-cell responses (28). In fact, it is believed that Treg may contribute to inefficient cell-mediated immunity on chronic HIV infection since some studies showed increased Treg frequencies in such phase (29–31). However, Treg may also have beneficial effects by limiting immune activation and, hence, restricting potential targets for HIV infection, as well as minimizing the pathological effects resulting from HIV-mediated immune hyperactivation (9). Such beneficial versus detrimental effects of Treg in HIV infection have been a subject of controversy in recent years (30, 32–35). In addition, the fact that Tregs are a subset of CD4+ T cells expressing CXCR4 and CCR5 coreceptors makes these cells susceptible of being infected by HIV, complicating even more the comprehension of the real role of Tregs in HIV infection. Recent findings from our group demonstrated that HIV can directly infect Tregs producing a marked deregulation on the phenotype and functionality of the cells (36, 37), and decreased Treg absolute counts described in infected patients (38–40) supports the hypothesis that Treg deregulation might be related to the immune hyperactivation present in HIV-infected patients.

The aim of this review is to provide a detailed scope of those mechanisms and molecules that are crucial for either Treg modulation or functionality but may also play a role in HIV infection (CD25, Foxp3, CD39/CD73, cAMP, ICER, and CTLA-4). The second part of the review will focus on how HIV direct infection of Tregs might jeopardize each of the mechanisms described in the first part by suppressing the Treg cornerstone molecules: CD25 and Foxp3. Finally, we will discuss how HIV-mediated Treg deregulation might contribute to the overall immune-hyperactivation scene characterizing HIV-infected patients and their clinical progression.

## Treg Immune-Modulation and Mechanisms of Action

### CD25/IL-2 Axis, the Master Regulator in the Maintenance of Balanced Immune Responses

Treg homeostatic role depends on the surface expression of the high-affinity interleukin 2 receptor alpha (CD25 or IL-2-Rα) and IL-2 uptake (41, 42), thus receiving the name of CD25/IL-2 axis. The CD25/IL-2 axis is essential for Treg survival, expansion, homeostasis and their suppressive function. Regarding the CD25/IL-2 axis role in Treg function, it is assumed that activated T cells are the main IL-2 producers; thus, there is a negative feedback control of immune activation through IL-2 availability (37). As soon as the number of IL-2 secreting T cells and IL-2 concentration increase, Treg will react via cellular expansion, uptaking the extracellular IL-2 and, thus, activating their suppressive function. Both Treg production of suppressive cytokines and IL-2 consumption by Treg are pivotal mechanisms to prevent an excessive T-cell expansion and to re-establish the homeostasis of the immune system (43, 44). This mechanism guarantees that the relative Treg:T-effector ratio is continuously maintained even though the number of CD4+ T cells is significantly altered (41, 43). It has been shown that the Treg capacity to sense IL-2 is directly responsible for their function and IL-2 availability is an important mechanism by which Tregs exert their role (44). In humans, *IL2RA* gene polymorphisms affecting CD25 function have been associated with multiple sclerosis, type 1 diabetes, juvenile idiopathic arthritis, or lymphoproliferative-associated immunodeficiency (43, 45), highlighting the dependency of Treg in this receptor to exert their function. Furthermore, CD25/IL-2 signaling through STAT5 is essential to sustain Forkhead box P3 (Foxp3) expression on Treg (46, 47), which is a critical factor to keep Treg fate and function (6, 48). The CD25/IL-2 axis also plays a critical role in cAMP production, being cAMP a crucial regulator of immune cells. It has been shown that Treg activation by IL-2 leads to a significant upregulation in the adenylyl cyclase (AC) activity and, hence, to the cAMP cytosolic accumulation (11). The high-affinity receptor, CD25, enables the Tregs to uptake extracellular IL-2 in advantage compared to other cells (41). IL-2 removal by Treg will avoid the IL-2-associated downregulation of AC isoform 7 (AC7) in conventional T cell and, therefore, the reduction of intracellular cAMP levels (11). Favoring low cAMP levels in conventional T cells is associated with an increase in T cell proliferation. The role of cAMP in immune response modulation will be extensively studied in following paragraphs.

In the context of HIV infection, CD4+ T cells undergo a marked activation followed by a status of exhaustion and senescence (49). It would be expected to find an increased production of IL-2 due to the extended T-cell activation, which should activate the Treg response to limit an excessive activation/expansion of effector T cells. However, there is evidence that this mechanism is not working properly since it is observed that the CD4+ T cell pool is permanently activated, becoming finally exhausted (50) and the immune activation will persist in HIV-infected patients. Moreover, it was already described a reduction in IL-2-producing cells in moderate and advanced stages of HIV type-1 infection (51). An explanation would be that IL-2 expression is repressed in CD4+ T cells during chronic HIV infection due to the increased methylation of IL-2 promoter observed in infected patients (52). In addition to its role in the Treg/effector balance, IL-2 has proven to inhibit HIV-1 replication in cell lines by the induction of APOBEC3G (53). Moreover, the therapy with recombinant IL-2 has been tested in HIV-infected patients with the goal of both to recover the CD4+ T cell counts and to mobilize the reservoir of latent virus activating the latently infected CD4+ T cells (54–56). However, despite a sustained increase of the CD4+ T cells count, these clinical trials involving recombinant IL-2 plus antiretroviral therapy (ART) did not show any clinical benefit (57). This highlights that there are many factors involved and the modification of IL-2 is not enough to control the fate of the disease.

All that points out the relevance of a deregulation in the CD25/IL-2 axis as one of the mechanisms related to the immune imbalance and subsequent hyperactivation found in HIV-infected patients.

### Foxp3, a Determinant Factor of Treg Identity and Functionality

Foxp3 is a crucial transcription factor determining Treg identity, development, and maintenance (6, 48). Expression of Foxp3 can also be induced and converts conventional CD4+ T cells into induced Treg cells (iTreg) (6). This iTreg generation could be observed in periphery or *in vitro*, and is induced by T-cell receptor (TCR) stimulation in the presence of IL-2 and TGF-β. Even though iTreg may be able to exert suppressive function as natural Treg, their Foxp3 is not stabilized by epigenetic modifications and, therefore, its expression is finally lost *in vivo* (58). Decreased Foxp3 expression in Treg is related to the switch to a cytokine-secreting profile characteristic from other CD4+ T cell helper lineages (48). Indeed, severe attenuation or ablation of Foxp3 expression resulted in the acquisition of the ability to produce effector cytokines, such as IL-2, IL-4, IL-17, TNF-α, and IFN-γ (48), and correct Foxp3 expression will suppress Th17 differentiation by inhibiting the function of the Th17 lineage-specifying transcription factor retinoid-acid receptor-related orphan receptor (ROR)-γt (59). Moreover, as transcription factor, Foxp3 can regulate gene expression of several genes, between which can be found CD39, adenine cyclase 9, and many others. Within its regulation role, it is interesting to note that Foxp3 forms a cooperative complex with NFAT, which is required to repress the cytokine IL-2 transcription and for upregulation of the cytotoxic T lymphocyte-associated antigen 4 (CTLA-4) and CD25 expression (60–62), both relevant markers for Treg function. In HIV context, Foxp3 transfection demonstrated inhibitory effects upon HIV transcription in primary human CD4+ T cells (63, 64). HIV transcriptional downregulation could be associated with NF-κB-dependent and -independent mechanisms (63), as well as to the Foxp3-dependent inhibition of NFAT activity. It was shown that Foxp3 decreases the binding of NFAT2 to the HIV promoter *in vivo* by its capacity to sequestrate it (61, 64).

Several articles describe increased relative frequencies of Tregs (CD4+ T cells expressing Foxp3) in HIV-infected patients (29, 65). This increased Treg proportion could be explained by a preferential depletion of conventional CD4+ T cells or by an increased expansion and/or induction of Tregs (65). Due to the absence of appropriate markers to distinguish natural from induced Treg, it is difficult to know whether the inflammatory or hyperactivated context of HIV infection promotes the differentiation of conventional CD4+ T cells to iTreg. Jenabian et al. recently demonstrated that soluble CD40-ligand (sCD40L) levels are increased in plasma from HIV-infected patients, and this sCD40L induced Treg expansion and favored Treg differentiation from conventional CD4+ T cells (66). Since iTregs are not able to maintain a long-term Foxp3 expression and suppressive activity, and since they can even acquire a pro-inflammatory phenotype, further studies on the Treg dynamics are required to understand the role of iTregs in the context of HIV infection.

### Extracellular ATP Metabolism and Signaling in T Cells: CD39/CD73 Axis

From among all the Treg mechanisms related to their suppressive capacity, adenosine triphosphate (ATP) metabolism is one that is well documented. In this context, there are two essential players that constitute the CD39/CD73 axis. CD39 or nucleoside triphosphate diphosphohydrolase 1 (NTDPase 1) is an ectoenzyme that hydrolyzes ATP or ADP to AMP (10). This enzyme is expressed by a subpopulation of Treg and, orchestrated together with another ectonucleotidase named AMPase CD73 present on the Treg surface, they are able to process AMP into adenosine (67). Adenosine exerts immune inhibitory effects as discussed in following paragraphs. It is interesting to note that Foxp3 expression is directly related to adenosine production since retroviral transduction of CD4+ CD25− lymphocytes with Foxp3 induced the expression of CD39 (6, 10), a potent inhibitor of cell proliferation and indirect contributor to the high cAMP levels found in Treg via adenosine generation (9).

In order to understand the formation of adenosine, we will describe the origin and relevance of ATP, which is the CD39/CD73 axis substrate. Extracellular ATP is released under hypoxia, inflammatory responses, metabolic stress, or other types of cell injury. The impact of extracellular ATP on the immune system is critical since its increase induces the activation of the inflammosome and subsequent release of cytokines, such as IL-1β (68, 69), in response to damage-associated molecular patterns (DAMPS) and pathogen-associated molecular patterns (PAMPS) (70). Therefore, extracellular ATP is considered a danger signal liberated by damaged or dying cells that induces pro- and anti-inflammatory signals. In the context of immune chronic activation as in HIV infection, ATP released by activated T cells seems to have an autocrine effect, prolonging activation and IL-2 secretion (71).

In contrast to ATP, adenosine exhibits anti-proliferative and inhibitory effects, hence giving to the CD39/CD73 activity an immune suppressive role (10). In fact, it was shown that induced Treg expressing CD39+ acquired higher suppressive capacity than CD39neg iTreg (72). Adenosine plays an antagonistic role on Treg compared to non-Treg responses by directly binding to the adenosine 2a receptor (A2AR), consequently inducing the adenylyl cyclase activity and, therefore, increasing the intracellular cAMP level. ATP removal and A2AR activation elicits inhibitory functions in dendritic cells and activated T-cell subsets, inducing T-cell anergy (73); whereas in Treg, A2AR induces the generation of Foxp3+ Tregs (73) and enhances Treg immunosuppressive mechanisms (74, 75). Summing up, Treg could dampen immune activation as well as induce activated T-cell dysfunction through CD39/CD73 activity.

It is interesting to note that a study of CD39/CD73 distribution in Treg and conventional CD4+ T cells showed that even though CD39 is largely expressed on human Treg (CD4+ CD25hiFoxp3+ T cells), CD73 is not so widely expressed and <1% of Treg expressed both ectonucleotidases at their surface (76–78). One hypothesis is that only few cells capable of hydrolyzing ATP to adenosine are necessary to induce a local suppression and that this pathway must be finely regulated to avoid any unwanted Treg-suppressive function. Moreover, it was described that CD73 was present in cytoplasmic granules and that its expression at the surface of Treg might be transient, which would be another level of regulation of adenosine production (77, 78).

In the case of HIV infection, there is high expression of CD39 in Treg cells, which remains unaltered even with ART (31, 79). CD39+ Treg frequency and number are elevated in HIV patients, and correlates negatively with CD4+ T-cell recovery and positively with plasma viral load and T-cell activation (80, 81). In addition, through a case-control study, a genetic variant of CD39 associated with lower expression of CD39 enzyme was linked to a slower progression to AIDS (81). Moreover, *in vitro* suppression assays demonstrated that the suppressive effect of CD39+ Treg upon gag-stimulated CD8+ cytokine production was partially inhibited when adding CD39 blocking monoclonal antibodies (81). All this may highlight a negative role of CD39+ Treg in controlling HIV progression. However, there is still controversy regarding the role of CD39 in HIV infection, Schulze et al. demonstrated that HIV controllers were shown to have similar CD39+ Treg to healthy subjects (79). Additionally, it has been shown that Foxp3 modulates the expression of CD39 at the surface of Treg and also regulates HIV promoter’s transcription activity. Moreover, CD39 might also be contributing to hindering HIV infection as suggested in following paragraphs.

In summary, Treg might have two contrary functions on HIV replication and disease progression. On the one hand, CD39 expression may be involved in Treg-mediated suppression of HIV-specific responses and, thus, on disease progression. On the other hand, Foxp3 induce a negative effect on HIV transcription, which could limit new particles production or induce HIV latency in Foxp3-expressing cells. However, one could claim that CD39+ Tregs may be critical for the inhibition of T-cell immune activation, which may reduce the niche for HIV replication (82, 83). In fact, blocking CD39 and, thus, inducing the decrease of cAMP levels in Treg were shown to abrogate the Treg-mediated suppression of HIV replication (84). Therefore, it is possible that CD39+ Foxp3+ Tregs could control HIV infection, especially during the first days of infection, prior to the HIV dissemination to the secondary lymphoid organs, phase where just a few effector T cells are activated (84).

### CD38 and NAD^+^ as Precursor of Adenosine

Few years ago, an alternate route to producing extracellular adenosine was discovered. This axis had its pivotal role around the NAD^+^-glycohydrolase CD38, the 5′-ectonucleotidase CD73 and the ecto-nucleotide pyrophosphatase/phosphodiesterase (PDE) CD203A (85). Leaded by CD38, main NAD^+^ consuming enzyme (86), it has been shown that CD38/CD203A/CD73 will transform NAD^+^ into adenosine (85), whose function is extensively described in this review. Horenstein et al. and others demonstrated in different *in vitro* models that this alternate pathway may synergize, flank, or bypass the aforementioned described canonical catabolic pathway orchestrated by CD39 (85–88). Regarding to the Treg population, it has proven to express CD38 (89, 90) and seems that its level of expression positively correlates with their suppressive function (90). Patton et al. demonstrated in a murine model that CD38^high^ Tregs have higher suppressive capacity than CD38^low^ Treg, which failed to upregulate CD73, key molecule for adenosine production, in both canonical and CD38 pathway. However, it is still under evaluation if Tregs are capable of processing NAD^+^ through this newly discovered pathway (85, 91).

Regarding to the role of CD38 in the context of HIV infection, this enzyme is commonly regarded as a T-cell activation marker, and peripheral blood CD38+ CD8 T cells have been strongly correlated with disease progression in untreated HIV infection (92, 93). It has been shown that rectal Treg frequency is positively related to CD38+ CD4+ and CD8+ rectal T cells in chronic HIV-positive non-controllers (94). However, this may vary depending on the sampling site and time point of infection, since Tregs are inversely correlated to CD38+ CD8+ T cells in blood at primary HIV infection as others have shown (95). Summing up, CD38 is not only a predictive marker of disease progression but also may be another mean for Treg to produce adenosine and exert its suppressive function.

### ICER and cAMP, Differential Role on CD4+ T-Cell Subsets

Cyclic adenosine monophosphate is a derivative of ATP. cAMP acts as a secondary messenger involved in many biological processes and in regulation of immune cells, playing an important role on determining the balance between suppression and activation. In fact, cAMP present on conventional T cells or on Treg is implicated in different signaling pathways.

#### cAMP Is a Major Mediator of Treg-Suppressive Potential

In Treg, the suppressive potential has been demonstrated to depend on cAMP (96). Indeed, Tregs tend to preserve their high levels of intracellular cAMP. To partially explain the maintenance of such high levels, it has been shown in murine nTreg cells that the expression of the cAMP degrading PDE3b is reduced by direct binding of Foxp3 to the PDE3B promoter (97), limiting Treg intracellular cAMP degradation. Treg can induce the intracellular cAMP production by the sequential processing of extracellular ATP to adenosine by the CD39/CD73 axis, inducing adenylyl cyclase activity in the target cell through A2AR (69, 75, 78). The importance of A2AR in mediating the inhibition of inflammation was demonstrated in A2AR null mice, where the transgenic animals suffered of massive tissue destruction and systemic inflammation due to their inability to control inflammation (98). It is interesting to note that A2AR engagement using an agonist not only was able to induce TGF-β and Foxp3 expression on activated T cells (73) but was also able to increase the intracellular cAMP level on purified Treg. Therefore, A2AR engagement increases the number of Tregs, activates their immunoregulatory activity, and allows iTreg generation. Thus, it has already been described how IL-2R, A2AR activation, and Foxp3 expression contribute to the “supraphysiological” intracellular levels of cAMP found in Treg. Treg can mediate cAMP transfer to activated CD4+ T cell and dendritic cells through gap junction intercellular communications (GJICs) (75, 96, 99). The formation of such channels composed by two opposing hemichannels allows the connexion between adjacent cells (100), therefore increasing intracellular cAMP in the target cells (96). In the case of DCs, cAMP induces the rapid downregulation of the co-stimulatory molecules CD80 and CD86, preserving their tolerogenic phenotype and indirectly regulating T-cell function (101). Furthermore, cAMP induction in DCs, following activation of adenosine and prostaglandin receptors, downregulates NF-κB activity not only decreasing the DC production of cytokines, such as TNF-α, IL-6, IL-12, IL-1α, and chemokines MCP-1 and MIP-1α, but also increasing the production of the anti-inflammatory cytokine IL-10 (102).

#### cAMP and ICER, Key Modulators of Conventional T-Cell Function

On conventional T cells, A2AR expression is induced after TCR stimulation via NF-AT-signaling pathway (73). This upregulation of A2AR expression was followed by an increase in intracellular cAMP level, demonstrated by the use of an A2AR-specific agonist (73). cAMP activation triggers a number of downstream signaling cascades on conventional CD4+ T cells. The main stream pathway is the activation of the cAMP-dependent protein kinase-A (PKA), which is involved in the regulation of several physiological processes as modulation of ion channels and transcription of genes (103). PKA will activate the transcription factor CREB by phosphorylation, consequently triggering the interaction of phospho-CREB and the nuclear protein CBP, which is a coactivator that augments the activity of phosphorylated CREB. Afterwards, CREB binds to a DNA element known as the cAMP-regulated enhancer element (CRE) activating the transcription of cAMP-responsive elements (104). Thus, consequently triggering the formation of the induced cAMP early repressor (ICER), which is generated from the 3′ region of the gene encoding the cAMP response element modulator (CREM) due to alternative promoter binding (105). ICER will bind to CRE and thereby represses the activity of its own promoter, constituting then a negative auto-regulatory loop (105). In fact, ICER was considered a master regulator of the cAMP response, inhibiting T-cell function, most specifically downregulating IL-2 production on effector T cells, critical for T-cell growth (12, 106). When ICER is transgenically over-expressed in mice T cells, these lymphocytes present proliferative defects hallmarked by decreased IL-2 production (13), similar to the effect on IL-2 production resulting from the co-culture of CD4+ T cells and nTreg (12). In parallel, when intracellular cAMP levels rise on effector T cells, NFAT/ICER complexes are formed; this will play an important role on suppressing NFAT activity via its sequestration. Therefore NFAT will not be accessible for the cytokines and chemokines transcriptional induction, such as IL-2, IL-4, TNF-α, IL-13, MIP-1α, MIP-1β, and GM-CSF (107, 108). Taken together, all this information highlights the critical role of ICER induced by cAMP transferred from nTreg and which mediates suppression of several immune mediators, especially IL-2 production on effector T cells (109) and, hence, demonstrates the relevance of the cAMP/PKA pathway in suppressing T-cell responses.

#### HIV Enhances Intracellular cAMP, a Double-Edged Immune Sword

It has been shown that intracellular cAMP levels of T cells from HIV-infected patients are higher than those from healthy subjects (110, 111). *In vitro* studies corroborated the presence of higher cAMP levels in HIV-infected primary T cells and T cell lines compared to uninfected cells (111, 112). Interestingly, HIV infection was not required to increase intracellular cAMP, since the T cell treatment with the envelope glycoprotein gp120 was already sufficient to elicit such response and activate cAMP/PKA pathway through Treg function (113, 114). However, the mechanisms underlying gp120-mediated response are still unresolved. The potential benefit or hazard of intracellular cAMP in the context of HIV infection is still a matter of debate.

It has been shown that cAMP/PKA pathway can inhibit HIV-specific T-cell responses and such inhibition will favor viral replication. Besides inhibiting immune responses, HIV induces impairment on proliferation in CD4+ and CD8+ T-cell subsets, through the activation of the cAMP/PKA pathway by viral proteins. In fact, the pre-treatment of the cells with a PKA inhibitor prevented the induction of anergy (112), highlighting the negative role of high intracellular cAMP resulting from HIV infection. In a similar fashion, CD39+ Tregs may be involved in the suppression of HIV-specific T-cell responses through the production of adenosine and consequent rise of cAMP in conventional T cells as shown by Nikolova et al (81). Similar to the PKA inhibitor experiment, it was demonstrated that the use of adenosine deaminase that hydrolyzes adenosine permits to revert the inhibition of immune responses and enhances the HIV-1-specific effector responses in an *ex vivo* model (115). On the other hand, Tregs could exert a direct inhibition of HIV infection and replication in conventional T cells by cell-to-cell contact. This mechanism is cAMP dependent and involved the PKA activation pathway (84). Therefore, cAMP may also play a positive role by inhibiting viral replication. In fact, there are a number of examples illustrating cAMP benefits; Banas et al. showed in infected monocytes and T-cell lines that the elevation of intracellular cAMP activates CREB protein that will compete for limiting amounts of CBP/p300 thereby mediating a repressive effect on HIV LTR/enhancer transcriptional activity (116). Furthermore, the use of *rolipram*, a PDE4 inhibitor, prevented the degradation of cAMP consequently inhibiting the transcription of the genes under the control of HIV-LTRs (117, 118). Interestingly, the blockage of PDE4 activity also prevented the nuclear import of HIV DNA in memory T cells (118). cAMP may also limit viral infection by modulating the expression of the T-cell coreceptors needed for viral entry, indeed it was shown a downregulation of CCR5 and CXCR4 in a T-cell line using an agonistic-like anti-A2AR mAb that activated the cAMP/PKA pathway (119). Summing up, cAMP seems to play an important role on preventing HIV entry, DNA nuclear import, viral transcription, and viral replication. All this highlights the beneficial role of Treg on limiting HIV infection in conventional T cells since they remove ATP by generating adenosine and transfer high amounts of cAMP to non-Tregs through GJICs that subsequently limit HIV replication.

### CTLA-4

CTLA-4 is a negative immune-modulator receptor of the CD28 superfamily of immune-regulatory molecules (such as CD80 or CD86) expressed in Tregs and with clear involvement in HIV disease. Its suppressive mechanisms have been extensively studied (14–17). Of note are Sakaguchi’s findings, where he demonstrated that nTreg-specific CTLA-4 deficiency or CTLA-4 blockade using anti-CTLA-4, impaired or abrogated nTreg-suppressive function *in vivo* and *in vitro*, respectively (14, 16). This is possible since nTreg will interact through CTLA-4 with activated or CD80/CD86 expressing conventional CD4+ T cells and/or APCs, thus mediating suppression. It is claimed that such interaction may act synergistically with expression of ICER in conventional T cells mediated by Treg (12). On the other hand, CTLA-4 has been proved to be selectively upregulated in HIV-specific CD4+ T cells in HIV-infected patients, and its expression correlated positively with disease progression (49). Interestingly, HIV-gp120 protein enhanced Treg-mediated suppression through CTLA-4 upregulation (114). Such enhancement is probably mediated by cAMP/PKA pathway since cAMP itself was shown to induce CTLA-4 upregulation in the absence of full T-cell activation (120). In fact, it could be reasonable to assume that CTLA-4 may synergize with ICER since it will be also upregulated due to cAMP/PKA downstream activation. However, despite the evidence of CTLA-4 involvement in HIV infection, other study showed that blocking CTLA-4 with an anti-CTLA-4 antibody did not demonstrated any differences in the frequency of HIV-p24^gag^-conventional T cells when these cells were co-cultured with Treg (84). Therefore, CTLA-4 might not play a major role in the Treg-suppressive activity in HIV infection. Taking all this information together, CTLA-4 would not act directly on the HIV replication, but would act through the suppression of HIV-specific CD4+ and CD8+ T cells function. Additionally, the presence of HIV or cAMP that induces CTLA-4 expression at the surface of Treg would increment this capacity.

## HIV Infection Impairs Treg Activity

### Susceptibility of Treg to Infection

Tregs express HIV-coreceptors CCR5 or CXCR4 at levels comparable to other CD4+ T cells (79), which renders Treg susceptible to HIV infection (36, 37, 121–123). Furthermore, naïve Tregs are capable of upregulating the membrane expression of CXCR4 and CCR5 upon TCR stimulation (122), increasing their susceptibility to infection. The two viral strains CXCR4 and CCR5-tropic HIV showed differential infection dynamics. We described *in vitro* that CXCR4 virus produce a stronger deregulation than CCR5-HIV in both phenotype and functionality when Tregs are infected (36). Other studies report that HIV-CCR5 infects Tregs at lower levels compared to Teff at both early and late points (121). By contrast, Tregs were more efficiently infected with CXCR4 strain compared to Teff at early time points, but the difference cleared at later time points of the virus life cycle (121). Whether Tregs constitute a major target for HIV or if they are more susceptible than conventional T cells to infection has been a matter of debate in the past years. However, recent *in vivo* studies have shown that circulating Tregs are not preferentially infected by HIV compared with effector T cells (121), in fact HIV infection induces deep cellular deregulation in CD4+ T cells, including the Treg subset. The HIV-mediated Treg deregulation affects the cell number, their phenotype, and functionality. During chronic HIV infection, the absolute Treg numbers in peripheral blood decline (40) even though the Treg frequency among the total CD4+ T-cell population is increased. In addition, our group has described that direct HIV infection of Tregs modifies the Treg phenotype and induces a strong impairment in their suppressive capacity (36). A similar outcome was observed in a study of HIV-infected patients with immune reconstitution disease after ART, they described that Treg exhibited reduced immunosuppressive capacity that was associated with over-active CD4+ T-cell responses (124). We also demonstrated *in vivo* an impaired capacity of Tregs to keep the balance between Treg/IL-2-producing cells in viremic HIV-infected patients due to direct Treg viral infection (37), and probably contributing to the immune hyperactivation observed in HIV patients.

These findings provide clarifying information to the debate concerning the beneficial versus the detrimental role of Tregs in HIV infection. Since the Treg number and function is impaired in the presence of HIV, the negative impact on the HIV-specific responses might be limited. However, Treg impairment has probably a higher impact in the absence of an adequate control of immune hyperactivation, which at present is the major factor related to the HIV disease progression in infected patients.

### HIV Downregulates Foxp3 Expression and Impairs the Suppressive Activity of Treg

We demonstrated that HIV infection of Treg modifies its phenotype and functionality through a process mediated by intrinsic methylation mechanisms (36), possibly in a Treg attempt to protect its genome against the expression of foreign DNA (125). Foxp3 gene expression is strongly downregulated in HIV-infected Treg, notably with CXCR4-tropic HIV, due to general increase in the CpG methylation pattern of two regulatory sites critical for Foxp3 expression in Tregs (36). It is known that DNA methylation in Treg can be addressed to two known DNA methyl transferases (DNMT1 and DNMT3b), since binding sites for DNMT1 and DNMT3b have been detected in Foxp3 locus (126). Interestingly, we have shown that the expression of DNMT3b was upregulated in HIV-infected Treg compared to non-infected Treg (36). Therefore, the DNMT3b increase might be responsible for the *de novo* methylation observed in Foxp3 promoter and, thus, be responsible for the subsequent downregulation of Foxp3 expression observed *in vitro* in HIV-infected Treg (36).

As already described, Foxp3 expression is critical for Treg-suppressive function (127). It was shown that *in vitro* HIV-infected Treg were less capable of suppressing the proliferation of T-effector cells in co-culture experiments (36, 128). These results support the impaired Treg-suppressive capacity that we observed in untreated HIV patients with high viral load (unpublished data). However, other studies claim that HIV does not impair Treg-suppressive function in patients (35, 129, 130) probably because they studied it in chronic infected patients, and it is known that the number of infected Treg is very low at the late stage of infection (121). Interestingly, the treatment of HIV-infected Treg with antiretroviral drugs, such as ZDV or T20, *in vitro* reverted the impairment of suppression since the loss of Foxp3 expression was avoided (36). Therefore, it seems that the Foxp3 downregulation may contribute to the Treg loss of suppressive function upon HIV infection. There are many suppressive mechanisms on Tregs susceptible to be affected by Foxp3 reduction as described previously. Foxp3 downregulation may have critical consequences on Foxp3-transcriptional program, being PDE3B gene the most repressed by Foxp3 (60, 127); therefore, its repression might be abrogated if Foxp3 is downregulated and could have detrimental effects upon Treg. In fact, it was shown by Gavin et al. that retroviral gene transfer and expression of PDE3B into Tregs was deleterious to Treg homeostasis (127). Such detrimental effect consisted in a significant reduction of PDE3B-expressing Treg numbers due to cell death or inefficient expansion, and it is most probably related to decreased cAMP intracellular levels due to its degradation by PDE3B (127). Other mechanism related to Foxp3 repression is associated with miR-142-3p transcriptional regulation (131). Foxp3 downregulates the miR-142-3p in order to keep the AC9/cAMP active in Treg since miR-142-3p was described to target AC isoform 9 expression, thus, reducing the intracellular cAMP levels. Therefore, downregulation of Foxp3 expression will lead to miR-142-3p expression and this will have an indirect effect on Treg cAMP levels. Therefore, miR-142-3p might potentially contribute to the decrease in Treg absolute numbers and Treg function impairment shown in HIV, associated with the inability to maintain high cAMP levels. Besides this mechanisms affecting overall cAMP levels, it has been shown that the repression of Foxp3 using siRNA in hepatocellular carcinoma patients induces a downregulation of CTLA-4 in those Treg with lower Foxp3 levels, compromising their function (132). Thus, similar outcomes may be expected from HIV-infected Treg whose Foxp3 gene is downregulated.

Besides transcriptional regulation, it is known that Foxp3 can also be regulated by various post-translational modifications, such as acetylation, ubiquitination, and phosphorylation, thus affecting its activity (133, 134). A decade ago, Li et al. and others showed that the transcriptional repression by Foxp3 involves a histone acetyltransferase (HAT) complex that includes HAT TIP60 and class II histone deacetylase 7 (HDAC7) (135, 136). Within this complex, TIP60 is the principal responsible for Foxp3 acetylation and Foxp3-mediated transcriptional regulation *via* STAT3 *in vivo* (136). Therefore, the association of HDAC7 and TIP60 to the Foxp3 N-terminal domain resulted to be a requirement for Foxp3-mediated transcriptional repression in terms of suppression of IL-2 production in T cells after TCR stimulation (136). In the same line as the previous comment, there are a number of animal models such as the TIP60 conditional knock out mice supporting the relevance of Foxp3 complex formation (133). Indeed, several studies showed that the failure of Foxp3 to associate with TIP60 and HDAC7 leads to altered Foxp3-dependent transcription (higher promoter activity and production of IL-2) and epigenetic modifications, limiting nTreg function at inflammatory sites and decreasing the Treg population in peripheral immune organs, consequently resulting in accelerated fatal autoimmune disease (133, 136, 137).

**Figure 1:**
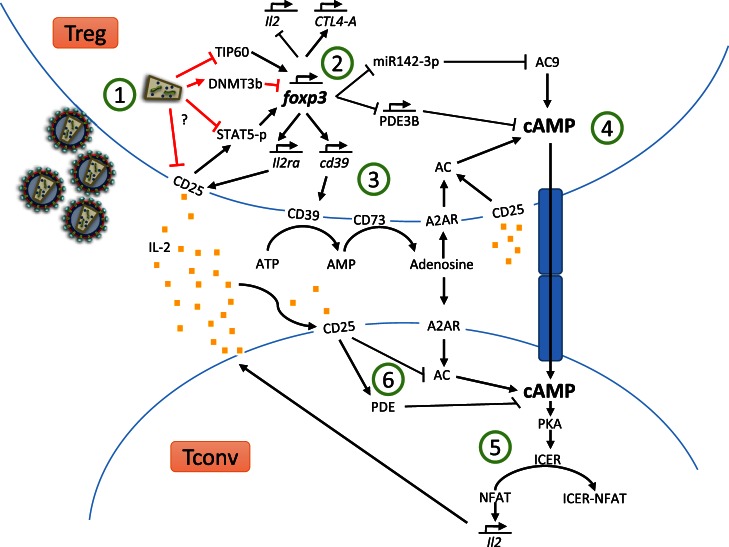
**Treg suppressor mechanisms and HIV-dependent Treg dysregulation**.

In terms of HIV infection, it has been shown that HIV-1 transactivator Tat protein represses TIP60 activity (138). Indeed, Tat induces the poly-ubiquitination and, thus, the degradation of TIP60 by the p300/CBP-associated E4-type ubiquitin-ligase activity (139), consequently affecting the Foxp3–TIP60–HDAC7 complex formation. Therefore, it could be expected a limited Treg function or decreased Treg levels as shown in the animal models (133, 137) (See Figure 1). In fact, the HIV-dependent disruption of Foxp3 complex may be contributing to the low Treg absolute numbers detected in HIV patients (140, 141).

Finally, HIV infection also leads to a disturbance on interleukin secretion profile (36) probably associated with Foxp3 downregulation (48, 142). Indeed, HIV-infected Tregs have shown a significant increase in IL-4 production and a decrease in TGF-β production (36). Since Foxp3 has a deep influence on Treg-associated genes expression (62) and that Foxp3 expression is highly modified during HIV infection (36), we can assume that such dysregulation in the production of cytokines may affect Treg function, since this dysregulation correlates with Treg switch to a T helper type 2 cytokine profile that may contribute to the hyperactivation shown in HIV patients.

### Impaired Balance between Regulatory T Cells and IL-2 Producing CD4+ T Cells in HIV Infection

The preserved and constant ratio between Treg and the number of activated T cells constitutes a homeostatic mechanism ensuring that T-cell activation and expansion remain under control (41). However, the persistent immune hyperactivation that drives HIV disease suggest that there is a dysfunction on such mechanism, since the Treg values are decreased despite high levels of activated T cells (34, 123, 140). In fact, a recent study indicates that the balance between Treg and IL-2 producing T-cell numbers is deeply disturbed in viremic HIV-infected patients (37). Such balance impairment is motivated by a HIV-mediated downregulation of IL-2 receptor alpha (CD25) at the surface of Treg from viremic patients, as confirmed by *in vitro* experiments of Treg infection with HIV (37). Therefore, the lower expression of CD25 observed in Treg from infected patients could affect their capacity of expansion due to the impossibility of responding to extracellular IL-2 produced by effector CD4+ T cells. Furthermore, decreased CD25 expression may also reduce Treg-suppressive function. Indeed, it has been reported in healthy donors that a lower CD25 expression correlates with impaired suppressive capacity and lower capability to maintain Foxp3 expression (143). CD25 downregulation correlates with the reduction of STAT5 activation in HIV-infected Treg (37), being STAT5 a key player on CD25 activation downstream cascade. Loss of phospho-STAT5 has been shown to abrogate Treg-suppressive activity and to induce the downregulation of Foxp3 expression (144). STAT5 and CD25 genes contain differentially methylated regions, and many of these regions present DNA methylation-dependent enhancer activity (145). Therefore, STAT5 and CD25 expression could be controlled by DNA methylation as occurs with Foxp3, thus explaining their downregulation upon infection. Therefore, the downregulation of Foxp3 expression, the functional impairment of Treg (36), and the impairment of CD25 pathway (37) observed in Treg from infected patients could be critical in the immune hyperactivation that hallmarks HIV disease.

### HIV Modifies the Treg Cellular Transcriptome Following Infection

It was shown by using an ultrasensitive multiplexed *NanoString* gene expression assay that HIV-1 modifies the Treg transcriptome after infection (128). Infected and non-infected Treg formed two distinct clusters showing differential expression in a total of 23 genes (128). HIV-infected Tregs show downregulation of genes linked to TCR signaling (PTPRC, TCR, IKBKE, CD3E), which may affect Treg antigen recognition and activation, and CD27 and PPAR-gamma that are related to suppressor function and enhancement of Treg function, respectively (146, 147). Furthermore, many other genes involved in function and Treg lineage were downregulated (TRAF6, STAT5, ETS-1, LEF-1) (148–151) but also Treg function inhibitor genes were upregulated after infection (LTA; IL-18RAP, BCL6, IL-7) (152–155), probably contributing to the HIV-dependent Treg impairment. Moreover, LEF-1, a gene that contributes together with Foxp3 in controlling transcription in Treg (151), and STAT5a, essential for Foxp3 expression, were downregulated (36, 46). However, in the Angin et al. study, Foxp3 downregulation was not detected and suggested a possible destabilization of the master transcription factor by downregulation of ETS-1 (148). Despite of these results, in recent years, we have shown that indeed HIV infection downregulates Foxp3 expression (36). Summing up, HIV-1 infection seems to exert Treg deregulation through a number of pathways simultaneously. The transcription pattern modification of critical genes involved in Treg function will probably play a detrimental role allowing HIV-1 hyperactivation and pathogenesis.

## Mechanism Summary

In Figure 1, we have gathered most of the Treg suppressor mechanisms covered in the review and how HIV-dependent dysregulation of Foxp3, CD25, and STAT5 may hinder Treg function.

Upon viral entry and replication (Figure 1 ①), HIV-1 Tat protein induces the poly-ubiquitination and degradation of TIP60, consequently disrupting the Foxp3–TIP60–HDAC7 complex formation, critical for Foxp3 function. Furthermore, Tregs suffer a downregulation of CD25 expression and, hence, a reduction in phosphorylated STAT5 levels. Additionally, HIV infection leads to DNMT3b upregulation, this methylase will methylate two regulatory sites critical for Foxp3 expression, inhibiting Foxp3. Moreover, STAT5 is essential to sustain Foxp3 expression, its downregulation further compromises Foxp3 level. With a reduced expression of Foxp3 (Figure 1 ②), miR142-3p and PDE3B expression will not be downregulated; therefore, PDE3B will start to degrade intracellular cAMP and miR142-3p will block AC9 mRNA, inhibiting cAMP production. Furthermore, Foxp3 induces CD39 gene expression; therefore, the lack of Foxp3 could result in decreased levels of CD39 and, hence, lower conversion of extracellular ATP to adenosine (Figure 1 ③). Low levels of extracellular adenosine will result in decreased signaling through A2AR and, hence, less induction of AC activity and lower cAMP production in either Tregs or conventional T cells. After viral infection, Treg may not be able to maintain their cAMP supraphysiological levels (Figure 1 ④), hence transferring less cAMP to conventional T cells through GJICs compared to optimal conditions. Insufficient cAMP levels in conventional T cells upon cellular contact, together with the decreased endogenous production of cAMP (Figure 1 ⑤), will result in lower ICER production via PKA activation and, therefore, NFAT will maintain the induction of pro-inflammatory cytokines, such as IL-2. NFAT will also bind to HIV-promoter enhancing HIV genes transcription. HIV-infected Treg may not only be unable to suppress the activity and IL-2 production by conventional T cells, but also will not be able to sense an increase in extracellular IL-2 levels as a result of T-cell hyperactivation (Treg CD25 is downregulated), thus impairing Treg proliferation and suppressive response. In addition, CD25 downregulation renders Treg less capable of consuming IL-2, this means higher IL-2 availability for conventional T cells. IL-2 captured by conventional T cells induces activation of PDE and AC7 downregulation, thus leading to intracellular cAMP degradation. Low intracellular cAMP is related to increased T-cells proliferation and activation. As a result of these HIV-mediated alterations of regulatory mechanisms, the balance between activated T cells and Treg will be broken, compromising the homeostatic fine-tuned balance of the immune system.

## Concluding Remarks

Retroviruses, such as HIV, have coexisted with the human race for millions of years. By that, host species have evolved to protect themselves by different mechanisms, such as restriction factors, but also by modulating the immune system to reach a fine-tuned response against the virus and other pathogens. As a consequence, virus counter-evolution leads to an evolutionary arms race. This phenomenon is well illustrated in Leigh Van Valens’s Red Queen Hypothesis, where two evolutionary systems, such as host/pathogen are constantly developing in order to maintain its fitness relative to the system they are co-evolving with. In this review it has been described how Treg exert a number of immune-modulatory mechanisms capable of hindering viral entry, replication and transcription but also guaranteeing the immune system homeostasis. However, HIV is capable of infecting and abrogating Treg function by inducing the downregulation of key molecules essential to enable Treg-dependent suppression. Impairment of Treg immune-modulatory function and induction of hyper-immune activation, provide an immunological condition favorable for viral infection and replication. The fact that HIV might exert a suppressive effect upon Treg function help us to understand that Treg role might be detrimental for HIV fitness and disease progression. This hypothesis means that the beneficial role of Treg on preventing immune hyperactivation outweighs the detrimental effect of Treg upon HIV CD4+ and CD8+-specific responses, which has been a matter of controversy in recent years. Up to date, it has been extensively described by others the pros and cons of Treg immunomodulatory mechanisms in HIV-infected patients and its effect upon disease progression. However, the increasing evidence of HIV’s direct impact upon the Treg population adds an additional layer of complexity considering the dysregulation of HIV-infected Treg and the contribution of such alterations to the disease progression. Understanding the immune-regulatory mechanisms that are impaired or deregulated will facilitate to unveil the rationale behind the immune hyperactivation that hallmarks HIV disease. This will open new avenues to find potential targets that could restore the immune homeostasis and, hence, decrease the systemic immune activation and exhaustion that determine the disease progression.

## Author Contributions

JL-A, RC-R, and MP have all participated in the conception, discussion, and elaboration of this review.

## Conflict of Interest Statement

The authors declare that the research was conducted in the absence of any commercial or financial relationships that could be construed as a potential conflict of interest.
