# Corrigendum: Functional Mechanisms of Treg in the Context of HIV Infection and the Janus Face of Immune Suppression

**DOI:** 10.3389/fimmu.2018.00792

**Published:** 2018-04-19

**Authors:** Jacobo López-Abente, Rafael Correa-Rocha, Marjorie Pion

**Affiliations:** Laboratory of Immunoregulation, “Gregorio Marañón” Health Research Institute (IISGM), Madrid, Spain

**Keywords:** Treg, HIV-infections, immunology, immune regulation, ICER

Unfortunately it was missed to state that the co-author Rafael Correa-Rocha is as well a Corresponding author of the Review article.

The authors apologize for this. This error does not change the scientific conclusions of the article in any way.

The original article has been updated.

## Conflict of Interest Statement

The authors declare that the research was conducted in the absence of any commercial or financial relationships that could be construed as a potential conflict of interest.

